# EMVI as an independent predictor of recurrence and the role of chemotherapy in N0 colonic adenocarcinoma: retrospective Cox regression analysis (2015–2022)

**DOI:** 10.1007/s00384-026-05088-9

**Published:** 2026-02-11

**Authors:** S. Bhanderi, M. Delaney, H. Khan, R. O’Neill, A. Patel

**Affiliations:** 1 Surgical Trainee, West Midlands Deanery, Birmingham, UK; 2 Department of Colorectal & General Surgery, University Hospitals Coventry & Warwickshire NHS Foundation Trust, Clifford Bridge Road, Coventry, UK

**Keywords:** Colorectal Cancer, Chemotherapy, EMVI, Recurrence

## Abstract

**Purpose:**

Extramural venous invasion (EMVI) is a high-risk pathological feature in colorectal cancer, yet its role in guiding adjuvant chemotherapy in node-negative colon cancer remains uncertain. This study evaluates EMVI as a predictor of recurrence in patients undergoing colon cancer resection and investigates whether adjuvant chemotherapy affects recurrence in node-negative, EMVI-positive (N0/EMVI +) patients.

**Methods:**

A retrospective cohort study was conducted on adults undergoing surgery for colon cancer at a single UK cancer centre between 2015 and 2022. Patients with rectal tumours or metastatic disease at presentation were excluded. Cox proportional hazards models were used to assess predictors of recurrence. Kaplan-Meier survival curves were generated to visualise recurrence-free survival (RFS) stratified by EMVI and chemotherapy status.

**Results:**

Among 675 patients, EMVI was present in 361 (53%). EMVI was independently associated with increased recurrence (HR: 1.80, 95% CI: 1.14–2.84, *p*=0.011). In the N0/EMVI+ subgroup (*n*=124), chemotherapy was not significantly associated with reduced recurrence: partial chemotherapy (HR: 1.36, 95% CI: 0.30–6.20, *p*=0.69), full chemotherapy (HR: 1.53, 95% CI: 0.46–5.12, *p*=0.49). Kaplan-Meier analysis revealed five-year RFS of 80.9% for no chemotherapy, 60.6% for partial chemotherapy, and 41.6% for full chemotherapy (*p*=0.69). Survival differences were not statistically significant.

**Conclusion:**

EMVI is a predictor of recurrence in patients undergoing surgery for colon cancer. However, in node-negative patients with EMVI, chemotherapy was not significantly associated with improved recurrence-free survival. These findings highlight the need for larger, prospective studies to better define the role of EMVI in guiding adjuvant therapy in stage II colon cancer.

## Introduction

Colon cancer is one of the most prevalent malignancies worldwide, with significant morbidity and mortality [1]. Despite advances in surgical techniques and adjuvant therapies, the risk of recurrence remains a major challenge, particularly in patients with specific high-risk pathological features [2]. Extramural vascular invasion (EMVI), characterised by the invasion of cancer cells into blood vessels beyond the muscularis propria, has emerged as a potential key prognostic factor in colorectal cancer, as its presence may be associated with an increased risk of distant metastasis and poorer overall survival [3, 4].

While EMVI is becoming recognised as a marker of poor prognosis, its role in guiding adjuvant treatment decisions, particularly in node-negative (N0) patients, remains unclear. Current clinical guidelines, such as those from the National Institute for Health and Care Excellence (NICE) and the European Society for Medical Oncology (ESMO), provide recommendations for adjuvant chemotherapy in stage III (node-positive) colon cancer but offer more nuanced guidance for stage II (node-negative) disease [5, 6]. Specifically, ESMO guidelines suggest only considering adjuvant chemotherapy in stage II patients with high-risk features, including EMVI, though this practice is not uniformly adopted [6].

The advent of advanced surgical techniques, including robotic-assisted colonic resection, alongside improving enhanced recovery pathways, has introduced new dimensions to the management of colon cancer. These innovations may influence patient outcomes and potentially impact the decision-making process regarding adjuvant therapies [7–9]. However, the interaction between surgical modality, EMVI status, and the efficacy of adjuvant chemotherapy in node-negative patients is not fully understood.

This study aims to evaluate the prognostic significance of EMVI in predicting recurrence following curative resection for colon cancer and to explore the role of adjuvant chemotherapy in node-negative EMVI-positive patients. By examining these factors, we seek to contribute to the ongoing debate about optimising treatment strategies for high-risk colon cancer patients and to provide insights that may inform future clinical guidelines.

## Methods

### Study design and patient population

A retrospective cohort study was conducted at a single UK tertiary colorectal cancer centre, including all adults (≥ 18 years) undergoing curative surgery for colon cancer. Tumours were classified as colonic based on anatomical site and type of surgical resection. Rectal cancers were excluded if documented as such in operative or histopathology reports. This centre has a full complement of multidisciplinary professionals and also has an established programme of robotic-assisted colorectal resection. Exclusion criteria included patients with rectal tumours, metastatic disease at diagnosis, or those who underwent palliative resection.

### Data collection

A prospectively maintained database of all colorectal cancer patients was queried to obtain a list of all eligible patients who underwent resectional surgery between January 2015 and December 2022. Data were extracted from electronic health records, demographic information (age, gender, ethnicity), comorbidity status as quantified by the Charlson Comorbidity Index, surgical details (type of resection, approach), histopathological data (tumour stage, grade, lymphovascular invasion, EMVI status, nodal status, resection margin status), and follow-up data including recurrence and survival status. Where patients received adjuvant chemotherapy, data were collected on whether the planned regimen was partially or fully completed. Data were collated and managed using Microsoft Excel for Windows.

### Statistical analysis

Statistical analyses were conducted using R version 4.3.3 (“Angel Food Cake”). To evaluate the impact of EMVI on recurrence and the effect of chemotherapy in node-negative EMVI-positive patients, Cox proportional hazards regression models were used. Time to event was defined as the time from the date of surgery to either the date of documented recurrence or the last follow-up date. The first Cox model assessed the association between EMVI and recurrence, adjusting for key clinical and pathological variables, including age, Charlson Comorbidity Index, T-stage, N-stage, resection margin status, and chemotherapy course. The second model focused on the node-negative (N0) and EMVI-positive subset, evaluating the association between adjuvant chemotherapy and recurrence in an exploratory manner while adjusting for other clinical factors. This analysis was intended to be hypothesis-generating rather than definitive, given the expected limited number of events within this subgroup.

Kaplan–Meier survival curves were generated to visualise the association between EMVI status and recurrence, as well as the impact of chemotherapy in the N0/EMVI-positive subgroup. The log-rank test was used to compare survival curves between groups.

### Ethical considerations

This study was conducted in accordance with the principles of the Declaration of Helsinki. Due to the retrospective nature of the study, informed patient consent was not required. All patient data were anonymised and securely stored to maintain confidentiality and in line with institutional data governance policies.

## Results

### Demographics

**Fig. 1:**
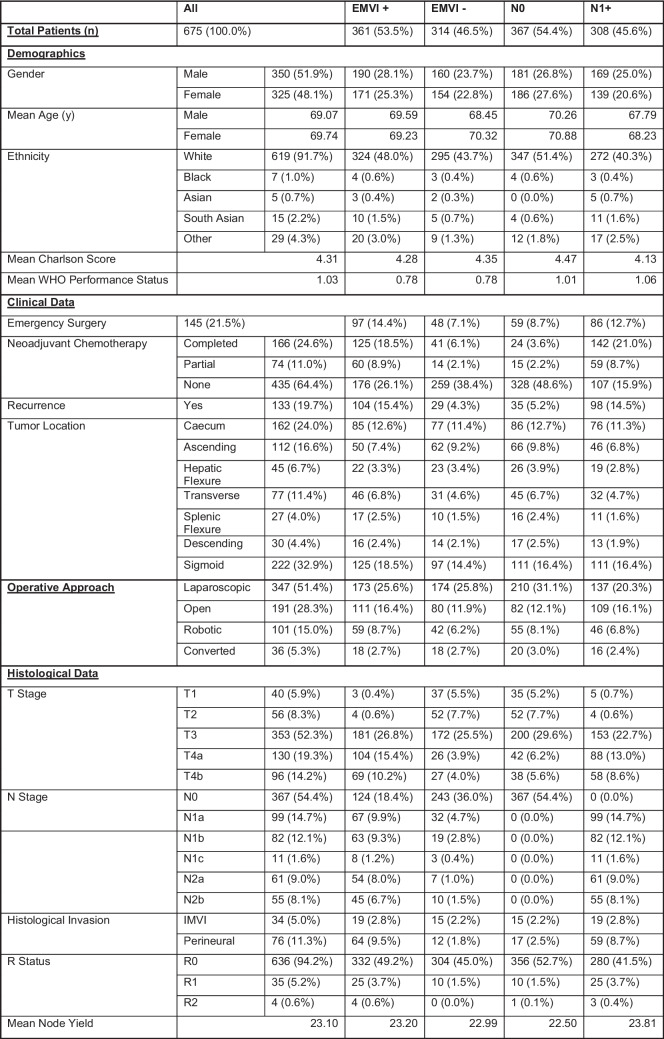
Summary table of demographic, clinical, histopathological and follow up data

A total of 675 eligible patients who underwent primary colon cancer resection with curative intent between 2015 and 2022 were included in the final analyses. The baseline characteristics of the patients are summarized in Fig. 1. The median age was 71 years (range 24–95 years), and 48.1% of the patients were male. Among the cohort, 361 (53.5%) were found to have extramural venous invasion (EMVI). Most patients (*n* = 435, 64.4%) did not receive adjuvant chemotherapy, while 74 (11%) received a partial course of chemotherapy and 166 (24.6%) received a complete course of chemotherapy.

### EMVI as an independent predictor of recurrence

**Fig. 2:**
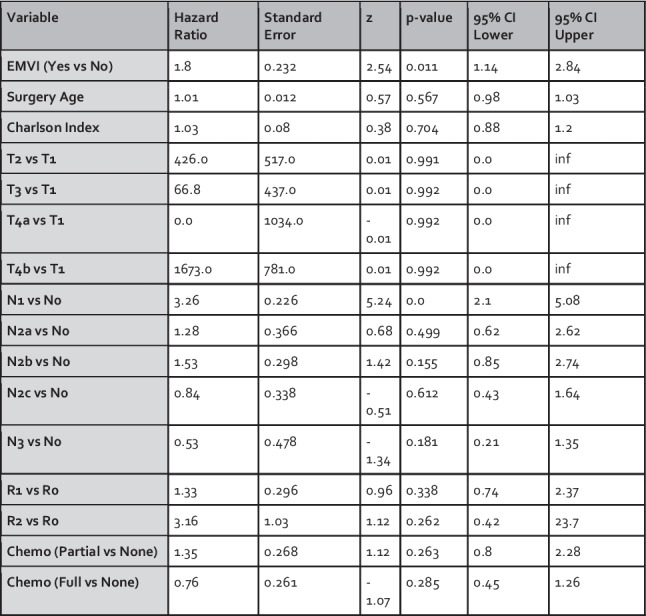
Cox proportional hazards model assessing predictors of survival for the overall cohort

A Cox proportional hazards model was applied to assess whether EMVI independently predicts recurrence in the overall cohort. The model included key clinical and pathological variables: EMVI status, Surgery Age, Charlson Comorbidity Index, T-stage, N-stage, R-status, and Chemotherapy Course. The results are presented in Fig. 2.

The analysis demonstrated that EMVI was independently associated with an increased risk of recurrence (HR: 1.80, 95% CI: 1.14–2.84, *p* = 0.011). N-stage was also significantly associated with recurrence (HR: 3.26, 95% CI: 2.10–5.08, *p* < 0.001). Other variables, including age, Charlson score, T-stage, R-status, and completeness of chemotherapy, were not significantly associated with recurrence.

Notably, several hazard ratios for T-stage comparisons (e.g., T2 vs T1: HR 426.0; T4b vs T1: HR 1673.0) were unusually large and accompanied by extremely wide confidence intervals. These results likely reflect statistical instability due to the very small number of patients with T1 tumours in the dataset (*n* = 40, 5.9%), with even fewer recurrence events occurring in this group. The low number of events within the reference group renders direct comparisons to it unreliable and inflates hazard ratio estimates.

**Fig. 3:**
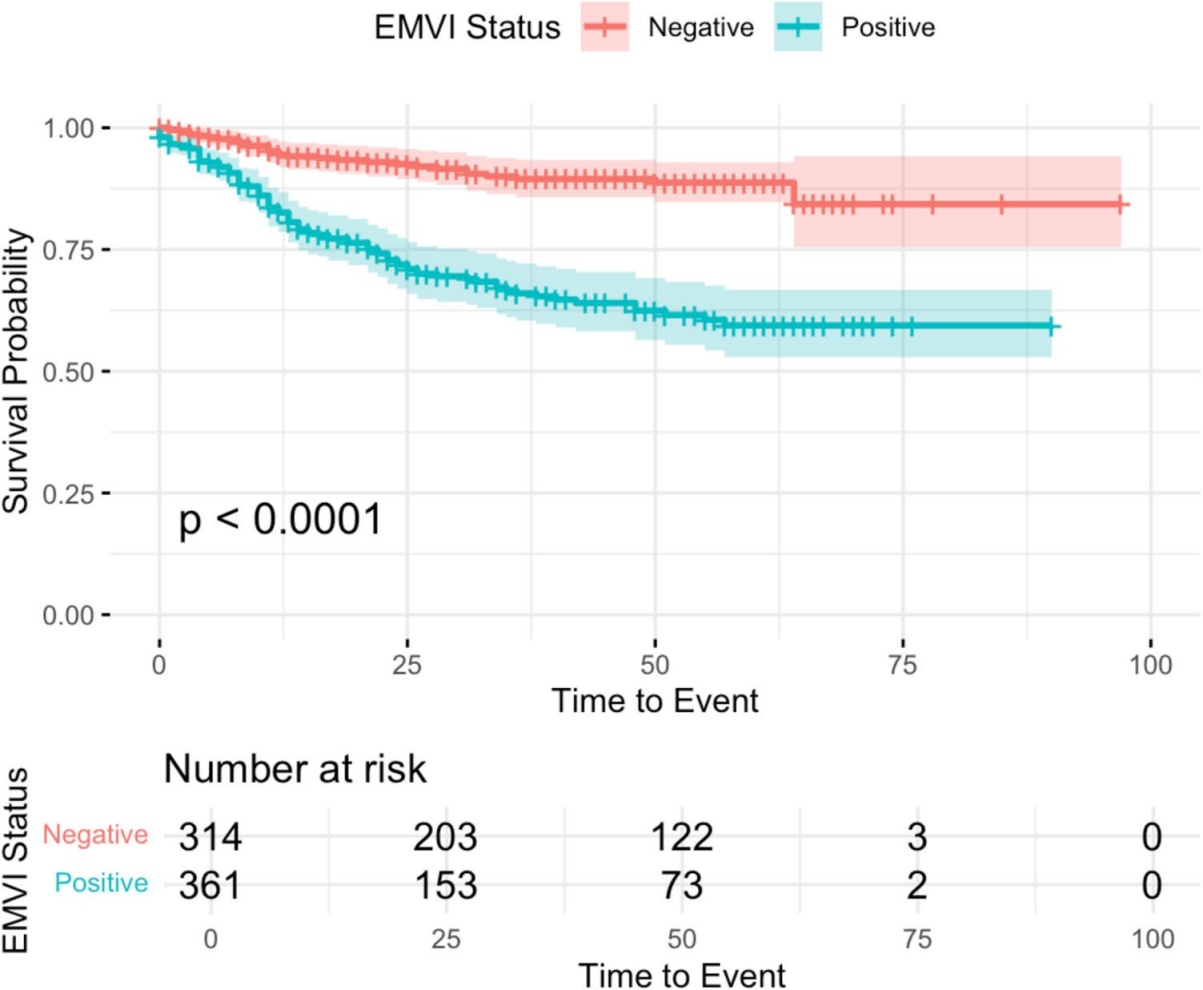
Kaplan Meier curve of recurrence against time for all patients undergoing curative colon cancer resection based on EMVI status

A Kaplan–Meier survival analysis further illustrated the impact of EMVI on recurrence. Patients with EMVI had a significantly lower recurrence-free survival compared to those without EMVI (*p* = 0.011), as shown in Fig. 3.

### Effect of chemotherapy on recurrence in N0/EMVI+ patients

To explore whether chemotherapy is associated with recurrence in node-negative, EMVI-positive (N0/EMVI+) patients, a subset analysis was performed using a Cox proportional hazards model. This included 124 patients who were both node-negative and EMVI-positive. The model assessed the impact of chemotherapy on recurrence, adjusting for gender, surgery age, Charlson Comorbidity Index, T-stage, and R-status.

**Fig. 4:**
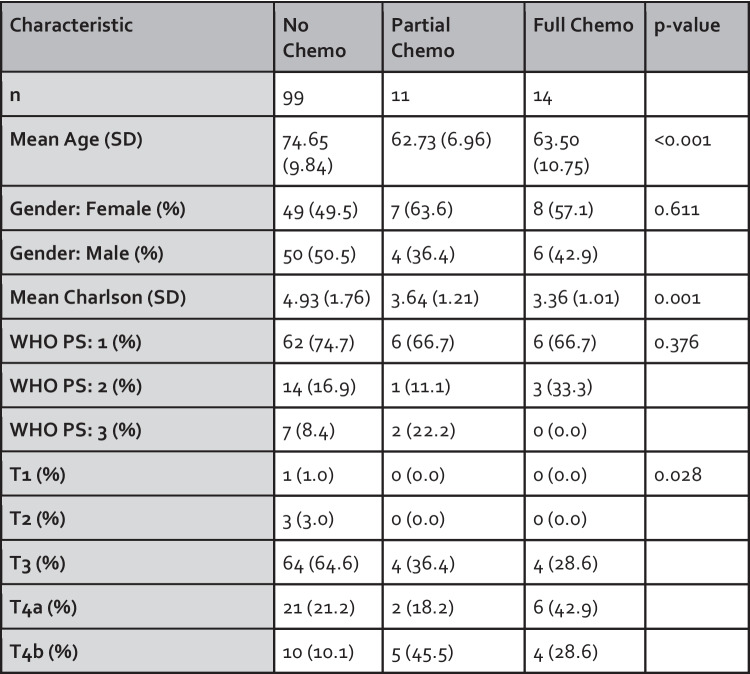
Demographic data for N0/EMVI+ patients across receipt of full course of adjuvant chemotherapy, partial or none

Figure 4 summarises baseline characteristics across chemotherapy groups (no, partial, and full chemotherapy). Comparisons were made using one-way ANOVA for continuous variables and the chi-squared or Fisher’s exact test for categorical variables, as appropriate.

**Fig. 5:**
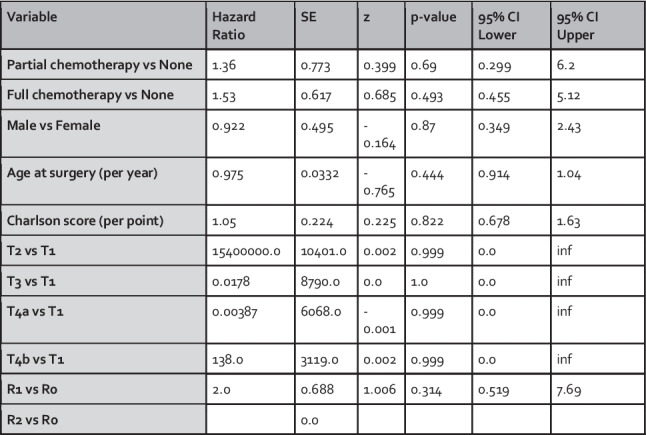
Cox proportional hazards model assessing predictors of recurrence in node-negative, EMVI-positive (N0/EMVI+) patients. (inf = infinity)

The Cox model revealed that neither partial chemotherapy (HR: 1.36, 95% CI: 0.30–6.20, *p* = 0.69) nor full chemotherapy (HR: 1.53, 95% CI: 0.46–5.12, *p* = 0.49) was significantly associated with reduced recurrence risk in this subgroup (Fig. 5). Hazard ratios for T-stage comparisons in this model also demonstrated marked instability and extreme values (e.g., T2 vs T1: HR > 15 million), consistent with the low number of recurrence events in the T1 reference group. As in the primary analysis, these results should be interpreted cautiously.

To explore whether combining partial and full chemotherapy groups might improve statistical power, a supplementary Cox model was constructed using a binary variable (any vs. no chemotherapy). This analysis also demonstrated no significant association between chemotherapy and recurrence-free survival (HR: 1.47, 95% CI: 0.48–4.53, *p* = 0.50).

**Fig. 6:**
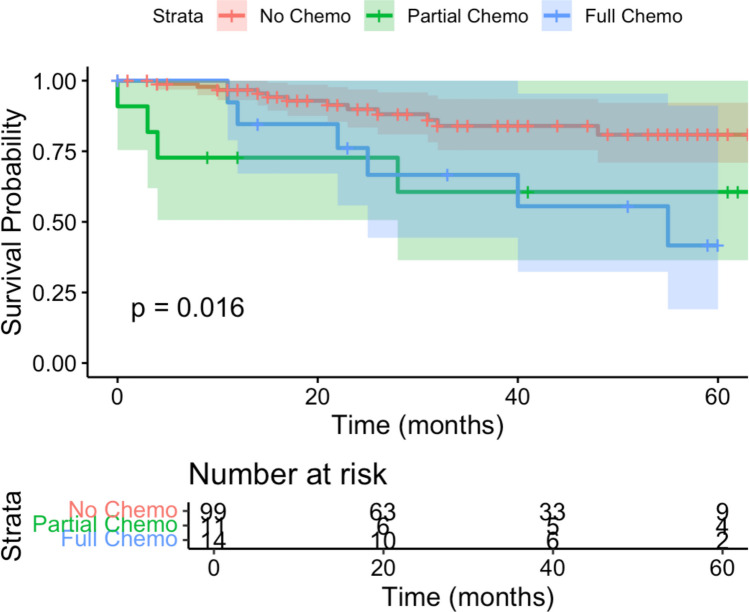
Kaplan Meier Survival Curves for EMVI+/N0 patients undergoing curative colon cancer resection, based on level of adjuvant treatment (no chemotherapy, partial chemotherapy, full chemotherapy)

Kaplan–Meier analysis further illustrated recurrence-free survival (RFS) by chemotherapy status. At five years, the estimated RFS was 80.9% (95% CI: 70.9%–92.1%) for patients who received no chemotherapy, 60.6% (95% CI: 36.4%–100%) for partial chemotherapy, and 41.6% (95% CI: 19.0%–91.1%) for full chemotherapy. There was no statistically significant difference between groups (*p* = 0.69), as shown in Fig. 6. However, interpretation is limited by the small sample size and low number of recurrence events within this subgroup.

### Effect of chemotherapy on node-positive patients

A short additional Cox proportional hazards analysis was performed on the subset of patients with node-positive colon cancer (*n* = 308) to explore the effect of chemotherapy on recurrence and confirm congruence with the current NICE/ESMO evidence base. Full chemotherapy was independently associated with a lower risk of recurrence (HR 0.54, 95% CI 0.31–0.93, *p* = 0.026), while partial chemotherapy was not (HR 1.04, 95% CI 0.60–1.81, *p* = 0.90).

## Discussion

This retrospective cohort study evaluated the prognostic role of EMVI in a contemporary cohort of patients undergoing curative resection for colon cancer with a UK cancer centre, and explored the association between adjuvant chemotherapy and recurrence in a specific subgroup of node-negative patients with EMVI positivity. Our findings demonstrate that EMVI is a significant predictor of recurrence, while chemotherapy did not show a statistically significant impact on reducing recurrence in the N0/EMVI + subgroup.

### Summary of key findings

Our study reinforces the critical role of EMVI as a prognostic factor in colon cancer, most likely due to its role in facilitating metastasis through vascular channels. EMVI positivity was associated with a significantly higher risk of recurrence, even after adjusting for other clinical and pathological variables. Although EMVI is an established adverse prognostic feature across colon and rectal cancers, there remains variability in its reporting, interpretation, and incorporation into adjuvant treatment decision making, particularly in N0 disease. By analysing a modern cohort treated in an era of routine multidisciplinary review, minimally invasive surgery, and contemporary pathological reporting standards, this study provides real-world evidence that supports the prognostic relevance of EMVI within current UK practice.

In contrast, our exploratory analysis did not demonstrate a statistically significant association between adjuvant chemotherapy and improved recurrence-free survival in N0/EMVI + patients. This suggests that the benefit of chemotherapy in this specific subgroup may be limited or influenced by factors not fully captured in our study.

### Comparison with clinical guidelines and literature

The National Institute for Health and Care Excellence (NICE) currently recommends adjuvant chemotherapy for stage III (node-positive) colon cancer but does not fully extend this recommendation to stage II (node-negative) colon cancer, reflecting the uncertainty in the benefit of chemotherapy in this group [5]. Nonetheless, the European Society for Medical Oncology (ESMO) guidelines suggest that adjuvant chemotherapy could be considered in node-negative patients who present additional high-risk features, including EMVI [6]. Our findings support the consideration of EMVI as a high-risk factor, although the specific role of chemotherapy in this context remains uncertain based on our data.

A recent 2023 analysis of 421 right hemicolectomies corroborates our findings, showing that EMVI + status is associated with poorer overall survival, independent of nodal status [10]. Interestingly, this study also suggested that when EMVI is absent, nodal status does not significantly influence survival, indicating that EMVI might be a more critical determinant of outcomes than previously understood. However, when EMVI is present, nodal status does influence survival, which adds complexity to decision-making regarding adjuvant therapy in these patients. McEntee et al. in 2022 also demonstrated an independent reduction in survival from colorectal cancer due to EMVI; however, their cohort included rectal cancers where EMVI has been more thoroughly scrutinised [11].

### Impact of surgical techniques and patient selection

Our study included patients who underwent open, laparoscopic, and robotic-assisted colonic resections. Studies indicate that robotic-assisted resection offers comparable long-term outcomes to laparoscopic surgery but may provide better short-term outcomes [12]. It might be that these short-term advantages, if present, could influence patient selection for adjuvant chemotherapy, potentially confounding the association between surgical technique and recurrence. Several previous single centre retrospective analyses have investigated the impact of minimally invasive resection on time to initiation of adjuvant therapy, with variations in findings. Although our study did not directly analyse these outcomes, the inclusion of various surgical approaches does improve the generalisability of our findings regarding adjuvant therapy and rates of recurrence. However, future studies, possibly with consideration of meta-analysis, should consider the impact of surgical technique on time course to adjuvant therapy.

### Strengths and limitations

A strength of our study is the comprehensive data collection from a high-volume cancer centre capable of various surgical techniques. This diversity in surgical approaches adds to the robustness of our findings. Additionally, our use of both Cox proportional hazards models and Kaplan–Meier survival analysis allows for a thorough examination of the factors influencing recurrence.

However, the retrospective design of our study introduces several limitations. The potential for selection bias is significant, as the decision to offer chemotherapy and the collection of data were based on medical records that primarily consisted of outpatient correspondence, which may not capture all relevant clinical details or nuances of decision-making processes. The relatively small sample size in the N0/EMVI + subgroup, coupled with a low number of recurrence events, limits the statistical power and generalisability of our findings. This also contributed to numerical instability in the Cox proportional hazards models, particularly when comparing recurrence risk between T-stage categories. Comparisons against the T1 reference group, which included very few patients and even fewer recurrence events, produced implausibly large hazard ratios with wide confidence intervals, reflecting the limitations of modelling with sparse event data. The COVID-19 pandemic, which overlapped with the data collection period, may also have influenced treatment decisions and patient outcomes. However, this inclusion reflects real-world clinical conditions and strengthens the external validity of our findings. Finally, we did not analyse the specific chemotherapy regimens administered, meaning potential differences in regimen efficacy were not accounted for. Overall, this analysis should therefore be interpreted as hypothesis-generating, and future prospective cohort studies will need to be large enough to ensure adequate event rates for robust analyses to inform any potential changes in practice.

### Conclusions and future directions

Our study underscores the importance of EMVI as a key factor in the risk stratification of colon cancer patients, suggesting that it could be a critical consideration in decisions regarding adjuvant therapy. However, the role of chemotherapy in N0/EMVI + patients remains uncertain, reflecting both biological heterogeneity and the absence of adequately powered prospective data. Our findings highlight this uncertainty and underscore the need for larger multi-centre studies to better define the role of EMVI in guiding therapy decisions for stage II colon cancer.

## Data Availability

The data that support the findings of this study are not openly available due to reasons of sensitivity and are available from the corresponding author upon reasonable request. Data are located in controlled access data storage at University Hospitals Coventry & Warwickshire NHS Trust.
